# The Impact of Lifestyle Counseling by Family Physicians on the Management of Hypertension in Primary Care Settings: A Systematic Review

**DOI:** 10.7759/cureus.93805

**Published:** 2025-10-04

**Authors:** Fahad Ali A Alsahli, Atheer Suliman Alanazi, Salwa Eiad Atassi, Saied Yaser Sharif, Rinad Yaser Sharif, Nour Eddin Imad Alzayed, Hassan Ali Albaggal, Ahmed Almalki, Rashed Mishary Alshammari

**Affiliations:** 1 Family Medicine, Hafar Al-Batin Health Cluster, Hafar Al-Batin, SAU; 2 General Practice, Vision College, Riyadh, SAU; 3 Medicine, Vision College, Riyadh, SAU

**Keywords:** blood pressure control, family physicians, guideline adherence, hypertension, lifestyle counseling, patient education, primary care, systematic review

## Abstract

Hypertension continues to be one of the most important avoidable risk factors for cardiovascular disease and early death. Many patients, especially in primary care settings, continue to have inadequate blood pressure control despite the availability of effective pharmaceutical medications. Fundamental tactics for avoiding and treating hypertension include dietary adjustments, greater physical activity, weight control, lowering salt intake, and quitting smoking. To evaluate the effectiveness of lifestyle counseling delivered by family physicians in primary care settings on the management of hypertension, a comprehensive search of four databases led to the discovery of 466 relevant publications. After eliminating duplicates and assessing each article for relevance, 28 full-text articles were examined, and ultimately, six studies were selected based on the inclusion criteria. Six studies were included, with a total of 7698 primary care physicians, and 4541 (58.9%) were men. The review included studies from multiple countries, most of which were cross-sectional in design. While a majority of physicians reported familiarity with hypertension guidelines, their actual application, especially in treatment and counseling, was often limited. Lifestyle counseling was inconsistently delivered, and adherence to clinical protocols varied widely. Outcomes showed that structured interventions and ongoing education improved physician performance and patient blood pressure control. The findings underscore the gap between hypertension guideline awareness and its real-world application. Enhancing training, embedding lifestyle counseling into routine care, and using patient-centered tools can significantly improve hypertension management outcomes in primary care.

## Introduction and background

Hypertension is a major modifiable risk factor for global cardiovascular morbidity and mortality, representing a leading cause of death within contemporary healthcare systems [1,2]. Its insidious onset, often remaining asymptomatic until significant end-organ damage has occurred, complicates early detection. The condition exhibits a high comorbidity with diabetes mellitus, with prevalence estimates ranging from 20% to 60%, influenced by variables such as body mass index, ethnicity, and age [3]. Globally, hypertension affects an estimated 1.28 billion adults aged 30-79 years, underscoring its significant public health burden [3].

Systemically, hypertension is clinically defined as a sustained elevation in blood pressure. According to the American Heart Association/American College of Cardiology guidelines, hypertension is diagnosed at an office reading of 130 mmHg systolic or 80 mmHg diastolic or higher [4]. Ambulatory blood pressure monitoring (ABPM) thresholds are correspondingly lower, with a mean 24-hour blood pressure of 125/75 mmHg or higher typically indicating hypertension [4].

The Seventh Joint National Committee emphasized that without a patient’s personal drive and involvement, treatment is bound to fall short. It is a bit like trying to sail a boat without the wind; no matter how well-built the boat is (i.e., the treatment plan), it will not move forward unless the wind (patient motivation) is there. Trust in the doctor’s approach and a genuine sense of care from the physician can act like wind in the sails, pushing the patient toward better outcomes [5].

In primary care settings, family physicians serve as the initial point of contact for a significant proportion of patients with hypertension. Their role is pivotal in the early detection of incident cases, the longitudinal management of established hypertension through pharmacotherapy, and the promotion of therapeutic lifestyle modifications. This comprehensive and proactive management strategy is essential, as evidence indicates that it can reduce the risk of major cardiovascular complications by at least 20% [6].

Hypertension remains one of the most significant preventable risk factors for cardiovascular disease and premature mortality worldwide. Despite the availability of effective pharmacological treatments, suboptimal blood pressure control persists in many patients, particularly in primary care settings. Lifestyle modifications, such as dietary changes, increased physical activity, weight management, reduced salt intake, and smoking cessation, are cornerstone strategies for both preventing and managing hypertension. Family physicians, as first-line healthcare providers, play a critical role in delivering lifestyle counseling and promoting long-term behavior change among patients. However, the consistency, effectiveness, and outcomes of such counseling interventions vary widely in practice. A systematic review is necessary to consolidate existing evidence and evaluate the true impact of lifestyle counseling by family physicians on hypertension management outcomes.

The objective of this systematic review is to evaluate the effectiveness of lifestyle counseling, encompassing key domains such as dietary modification, physical activity, and smoking cessation, delivered by family physicians in primary care settings for the management of hypertension. Specifically, it aims to assess the impact of such interventions on blood pressure control, patient adherence to lifestyle changes, and overall cardiovascular risk reduction. This review will also identify key components of successful counseling strategies and highlight potential barriers and facilitators to their implementation in real-world clinical practice.

## Review

Methods

This systematic review was conducted in accordance with the Preferred Reporting Items for Systematic Reviews and Meta-Analyses (PRISMA) guidelines to ensure methodological transparency and reliability [7]. The review aimed to synthesize current evidence on the impact of lifestyle counseling provided by family physicians on the management of hypertension in primary care settings, focusing on outcomes such as blood pressure control, adherence to lifestyle changes, and cardiovascular risk reduction.

Search strategy

A systematic literature search was conducted in accordance with the Preferred Reporting Items for Systematic Reviews and Meta-Analyses (PRISMA) guidelines. The electronic databases PubMed, Scopus, Web of Science, and Embase were searched. The search strategy was developed in collaboration with a medical librarian and utilized a combination of controlled vocabulary (e.g., MeSH in PubMed and Emtree in Embase) and free-text keywords.

The core concepts of the search were (1) hypertension, (2) lifestyle counseling, and (3) primary care/family physicians. These concepts were combined using Boolean operators. For instance, the PubMed search strategy includes (“Hypertension”[Mesh] OR “blood pressure” OR hypertens) AND (“Counseling”[Mesh] OR “Life Style”[Mesh] OR “health education” OR “behavioral intervention” OR “diet” OR “exercise” OR “smoking cessation”) AND (“Primary Health Care”[Mesh] OR “Family Practice”[Mesh] OR “general practitioner” OR “family physician”). This strategy was adapted for the syntax of each respective database. Furthermore, a manual search of the reference lists of all included articles and relevant systematic reviews was performed to identify any additional eligible studies.

Study selection and eligibility criteria

Two independent reviewers screened titles and abstracts, followed by full-text evaluation to determine eligibility. Any discrepancies in study inclusion were resolved through discussion or consultation with a third reviewer. The inclusion and exclusion criteria are summarized in Table 1.

**Table 1:** Inclusion and exclusion criteria

Category	Criteria
Inclusion	
Population	Adult hypertensive patients managed in primary care settings
Intervention	Lifestyle counseling (e.g., dietary advice, exercise guidance, and smoking cessation) delivered by family physicians
Outcomes	Reported clinical outcomes related to hypertension management
Study designs	Randomized controlled trials (RCTs), cohort studies, and cross-sectional studies
Publication period	Studies published from 2000 to 2025
Exclusion	
Study types	Case reports, narrative reviews, editorials, and conference abstracts without full data
Focus	Studies focusing solely on pharmacological interventions without a lifestyle counseling component

Data extraction was conducted by two independent reviewers using a standardized form. The extracted information encompassed various aspects, including study characteristics such as the author, year, country, study design, and sample size. Additionally, participant demographics were recorded, including age, sex, baseline health status, and the diagnostic criteria for hypertension. Details of the intervention were also noted, specifically the type, duration, and frequency of lifestyle counseling provided. Finally, the outcomes assessed included changes in systolic and diastolic blood pressure, adherence to the lifestyle advice, and any secondary cardiovascular risk indicators. Any discrepancies that arose during this process were addressed and resolved through consensus.

Risk of bias assessment

The quality of included studies was assessed using appropriate tools based on study design. For non-randomized studies, the ROBINS-I tool was applied to evaluate bias due to confounding, selection, and outcome measurement [8]. Two reviewers independently assessed each study’s risk of bias, and disagreements were resolved through discussion.

Results

**Figure 1:**
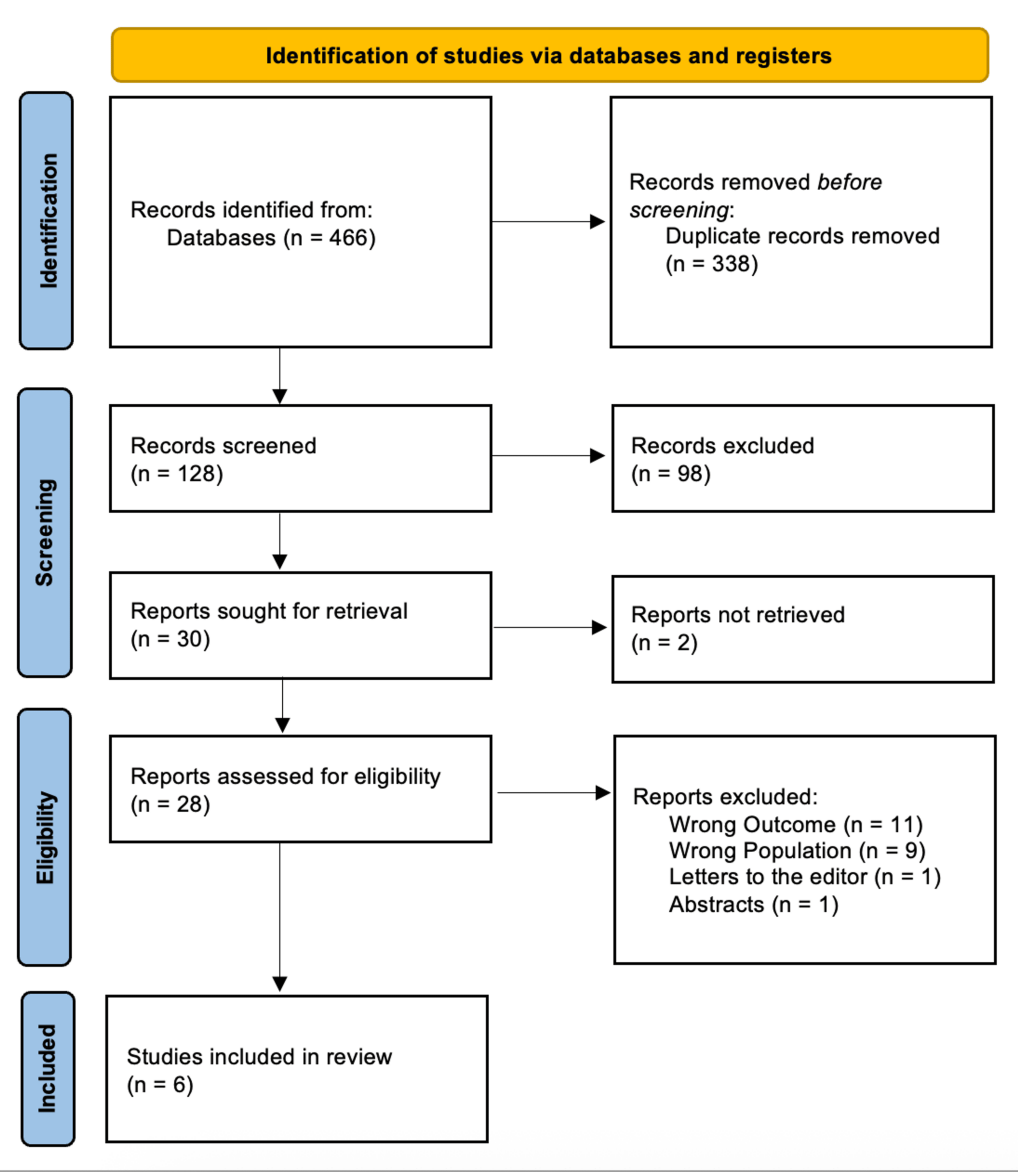
Search summary illustrated in PRISMA flowchart PRISMA: Preferred Reporting Items for Systematic Reviews and Meta-Analyses

The search process initially identified 466 publications (Figure 1). After removing 338 duplicates, 128 trials were screened based on their titles and abstracts. Of these, 98 did not meet the eligibility criteria, leaving 30 full-text articles for in-depth evaluation. In the end, six studies met the inclusion criteria and were selected for evidence synthesis and analysis.

Sociodemographic and clinical outcomes

Six studies were included, with a total of 7698 primary care physicians, and 4541 (58.9%) were men. The study designs consisted of six cross-sectional studies [9-14]. Two studies were conducted in Egypt [12,14], one in Canada [9], one in Kuwait [11], and one in Lithuania [13] (Table 2).

**Table 2:** Summary of demographics from the included studies MOC, Maintenance of Certification; ABP, ambulatory blood pressure

Study ID	Country	Study design	Sociodemographic	Hypertension management	Main outcomes
McAlister et al., 2000 [9]	Canada	Cross-sectional	Cases, 94; mean age: 45; men, 28 (39%)	The study highlights that current hypertension management guidelines, which often base treatment thresholds on physician or expert opinions, may not align with patient preferences. These guidelines typically recommend initiating antihypertensive therapy based on estimated cardiovascular risk levels	The study revealed significant variability in the minimum clinically important differences (MCIDs) between physicians and patients. Patients were generally more hesitant to accept antihypertensive treatment, especially at lower cardiovascular risk levels, and they required greater expected benefits before agreeing to therapy
Peterson et al., 2016 [10]	United States	Cross-sectional	Cases, 7319; mean age, 47; men, 4376 (59.8%)	The study illustrates how MOC programs can support quality improvement (QI) initiatives among family physicians managing hypertension. Over half of the participating physicians selected lipid control as their QI focus, suggesting a recognition of its importance in cardiovascular risk management	The findings advocate for the integration of MOC as a system-wide strategy to enhance clinical practices related to chronic conditions such as hypertension, which contribute significantly to morbidity and mortality
Al-Ali et al., 2013 [11]	Kuwait	Cross-sectional	Cases, 114; age range, 25-52; men, 36 (31.6%)	While most physicians agreed with and reported following the hypertension guidelines, their actual knowledge of the content was limited; only 27.2% answered at least 15 of 20 statements correctly	The study highlights a gap between familiarity with hypertension guidelines and their correct application among family physicians
Akl et al., 2006 [12]	Egypt	Cross-sectional	Cases, 27; men, 11 (40.7%)	The findings underline the importance of integrating holistic care principles into hypertension management and the role of continuous medical education (CME) in improving physician competency	While no physician expressed a negative attitude toward holistic care, variations in practice levels and knowledge suggest the need for targeted training interventions
Petrulionienė and Apanavičienė, 2010 [13]	Lithuania	Cross-sectional	Cases: 429 patients (29.3% were men)	Despite evidence-based recommendations favoring more intensive treatment for these groups, the data reveal suboptimal blood pressure control and inadequate therapy adjustments. The findings support the call for more rigorous monitoring and timely treatment modifications, especially when target ABP levels are not achieved	The study emphasizes the need for stricter adherence to hypertension treatment guidelines, particularly for patients at high and very high cardiovascular risk
Ahmad et al., 2022 [14]	Egypt	Cross-sectional	Cases, 120; men, 76 (63.3%)	Although 68.3% of physicians received training, only 56.7% used the guidelines in practice. Their knowledge was mostly fair, strongest in definitions and investigations but weakest in treatment. Adherence was partial, with high compliance in measuring blood pressure and history taking but low in treatment and clinical examination	The study underscores a significant gap between training and the actual application of hypertension management guidelines among primary healthcare physicians

Hypertension management

The included studies reveal varied approaches to hypertension management in primary care, often highlighting the gap between clinical guidelines and real-world practice. In some settings, hypertension guidelines were heavily reliant on physician or expert opinions and did not always align with patient preferences, particularly regarding thresholds for initiating therapy based on cardiovascular risk levels [9]. Maintenance of Certification (MOC) programs were identified as valuable tools to support quality improvement initiatives, especially when physicians focused on lipid control as part of cardiovascular risk management [10]. However, despite high reported agreement with hypertension guidelines, actual knowledge and application were limited. For example, a substantial portion of physicians failed to correctly answer key guideline content questions, with treatment protocols frequently being the weakest area of understanding [11]. Furthermore, integrating holistic care and continuous medical education was seen as essential to improving practice standards, particularly in environments where physician performance varied significantly by facility [12]. Suboptimal blood pressure control and insufficient treatment adjustments were prevalent, especially among high-risk patients, underscoring the need for more rigorous monitoring and therapy modification strategies [13]. Finally, the limited use of guidelines in practice, despite prior training, emphasized the need for enhanced implementation support and practical training in guideline adherence [14].

Main outcomes

Across the studies, several consistent outcomes emerged. There was notable variability in clinical decision-making between physicians and patients, with patients often requiring greater perceived benefit before accepting antihypertensive therapy [9]. MOC-based interventions led to improvements in counseling practices and blood pressure control, demonstrating their potential to enhance chronic disease management across specialties [10]. Nonetheless, a persistent disconnect between guideline familiarity and correct application was evident, with many physicians exhibiting only fair knowledge and partial adherence to core clinical practices, particularly in treatment and physical examinations [11,14]. Holistic care attitudes were generally positive, but the uneven levels of practice competency further reinforced the value of targeted training efforts [12]. The low adherence to intensified treatment strategies in high-risk groups and the continued use of monotherapy in uncontrolled patients pointed to significant clinical inertia, signaling a need for stricter adherence to evidence-based guidelines [13]. Overall, the findings highlight systemic gaps in translating hypertension management guidelines into consistent clinical outcomes and the necessity for integrated educational and quality improvement frameworks.

Table 3 shows that most studies exhibit low to moderate bias in various domains, indicating overall reliability in their findings.

**Table 3:** Risk of bias assessment using ROBINS-I

Study ID	Bias due to confounding	Bias in the selection of participants	Bias in the classification of interventions	Bias due to deviations from the intended interval	Bias due to missing data	Bias in the measurement of outcomes	Bias in the selection of reported results	Overall bias
McAlister et al., 2000 [9]	Low	Low	Moderate	Low	Low	Low	Moderate	Low
Peterson et al., 2016 [10]	Moderate	Low	Low	Low	Low	Moderate	Low	Low
Al-Ali et al., 2013 [11]	Low	Low	Low	Low	Low	Low	Moderate	Low
Akl et al., 2006 [12]	Moderate	Moderate	Low	Low	Low	Moderate	Moderate	Moderate
Petrulionienė and Apanavičienė, 2010 [13]	Moderate	Moderate	Low	Moderate	Low	Moderate	Low	Moderate
Ahmad et al., 2022 [14]	Moderate	Moderate	Low	Moderate	Moderate	Moderate	Low	Moderate

Discussion

This systematic review highlights the critical role of family physicians in delivering lifestyle counseling for effective hypertension management in primary care settings. The evidence reveals substantial variability in guideline adherence, clinical knowledge, and the implementation of counseling strategies across different regions and healthcare systems. Although many physicians express familiarity with hypertension guidelines, actual clinical application remains inconsistent, especially in the areas of treatment planning and patient follow-up.

Between 1986 and 1992, Canada reported notably low rates of hypertension treatment and control, with only 39% of patients receiving treatment and a mere 16% achieving control [15]. In contrast, data from the United States from 1988 to 2000 indicated comparatively better outcomes, with 58% of patients treated and 31% reaching blood pressure targets [16]. However, more recent data from a direct measurement survey in Ontario showed significant progress, with 81% of individuals receiving treatment and 65% achieving control [17]. These improvements, along with evidence from administrative studies showing a rise in physician-diagnosed hypertension cases [18] and a reduction in hypertension-related mortality over the past 10 years [19], indicate a substantial enhancement in how family physicians manage hypertension.

Bakris reported that family physicians play a vital frontline role in managing patients with both diabetes and hypertension by conducting annual screenings for cardiovascular and renal risk factors, educating patients on treatment goals, and guiding adherence to complex medication regimens. They initiate and adjust therapies, often starting with angiotensin-converting enzyme (ACE) inhibitors or angiotensin receptor blockers (ARBs), while managing concerns such as minor creatinine increases through safe interventions. By tailoring multidrug treatment plans and ensuring regular follow-up, family physicians significantly reduce the risk of cardiovascular and renal complications, aligning with national clinical guidelines and addressing the growing diabetes epidemic [20].

Furthermore, we found that while lifestyle counseling, such as dietary advice, physical activity promotion, and smoking cessation, is widely recognized as essential, its delivery is often underutilized or insufficiently emphasized in daily practice. Patient resistance to treatment, especially when perceived benefits are unclear, further complicates hypertension control efforts. These findings point to a persistent gap between knowledge and practice, which may contribute to the suboptimal control of blood pressure among hypertensive patients, particularly those at higher cardiovascular risk. Tu reported that family physicians are responsible for diagnosing, monitoring, and initiating treatment, often improving patient outcomes by following national guidelines such as those from the Canadian Hypertension Education Program. Despite challenges such as patient nonadherence and time constraints, they have significantly improved treatment and control rates. Their efforts in managing comorbidities, especially in diabetic patients, and promoting medication adherence make them key players in reducing the burden of cardiovascular disease [21]. Alharbi et al. also stated that family physicians deliver individualized care by initially detecting potential health issues through screening and then guiding patients in managing and adapting to their conditions. They offer advice on maintaining a healthy lifestyle and ensure that prescribed medications are appropriate. Additionally, they support patients by encouraging regular physical activities such as walking, running, or cycling to help manage their health [22].

Strengths

This review’s primary strength lies in its comprehensive and systematic approach, guided by PRISMA methodology, which ensured methodological rigor and transparency. It included studies from diverse geographical and clinical settings, providing a broad perspective on current practices in hypertension management by family physicians. The use of multiple databases and clearly defined inclusion criteria enabled a robust synthesis of existing literature. Additionally, the focus on both physician behavior and patient-related outcomes allowed for a well-rounded understanding of the dynamics influencing hypertension control.

Limitations

Despite its strengths, this review also has limitations. All the included studies were cross-sectional, limiting the ability to establish causal relationships between lifestyle counseling and clinical outcomes. There was also heterogeneity in how lifestyle counseling and adherence were defined and measured across studies, which may have affected comparability. Language and publication date restrictions may have excluded relevant data published in other languages or earlier timeframes. Lastly, variations in healthcare systems and cultural attitudes across countries may influence the generalizability of findings to other settings.

## Conclusions

In conclusion, this review affirms the pivotal role of family physicians in hypertension management through lifestyle counseling but underscores a critical need for better guideline integration. The findings suggest that adopting structured, evidence-based counseling frameworks, such as the five As (assess, advise, agree, assist, and arrange) and motivational interviewing (MI), can enhance efficacy in primary care. Ultimately, equipping providers with these skills, coupled with robust institutional support, is essential to overcome implementation barriers, achieve optimal blood pressure control, and reduce the global cardiovascular disease burden.
